# Cultivation and Genome Sequencing of Bacteria Isolated From the Coffee Berry Borer (*Hypothenemus hampei*), With Emphasis on the Role of Caffeine Degradation

**DOI:** 10.3389/fmicb.2021.644768

**Published:** 2021-04-06

**Authors:** Fernando E. Vega, Sarah Emche, Jonathan Shao, Ann Simpkins, Ryan M. Summers, Meredith B. Mock, Dieter Ebert, Francisco Infante, Sayaka Aoki, Jude E. Maul

**Affiliations:** ^1^Sustainable Perennial Crops Laboratory, United States Department of Agriculture, Agricultural Research Service, Beltsville, MD, United States; ^2^Sustainable Agricultural Systems Laboratory, United States Department of Agriculture, Agricultural Research Service, Beltsville, MD, United States; ^3^U.S. Department of Agriculture, Agricultural Research Service, Beltsville, MD, United States; ^4^Department of Chemical and Biological Engineering, The University of Alabama, Tuscaloosa, AL, United States; ^5^Department of Environmental Sciences, Zoology, University of Basel, Basel, Switzerland; ^6^El Colegio de la Frontera Sur (ECOSUR), Tapachula, Mexico; ^7^Department of Plant and Environmental Protection Sciences, University of Hawai’i at Mānoa, Honolulu, HI, United States

**Keywords:** bark beetle, broca del café, *Coffea*, Coleoptera (beetles), symbiosis, symbiotes, *Hypothenemus hampei*, coffee

## Abstract

The coffee berry borer, the most economically important insect pest of coffee worldwide, is the only insect capable of feeding and reproducing solely on the coffee seed, a food source containing the purine alkaloid caffeine. Twenty-one bacterial species associated with coffee berry borers from Hawai’i, Mexico, or a laboratory colony in Maryland (*Acinetobacter* sp. S40, S54, S55, *Bacillus aryabhattai*, *Delftia lacustris*, *Erwinia* sp. S38, S43, S63, *Klebsiella oxytoca*, *Ochrobactrum* sp. S45, S46, *Pantoea* sp. S61, *Pseudomonas aeruginosa*, *P. parafulva*, and *Pseudomonas* sp. S30, S31, S32, S37, S44, S60, S75) were found to have at least one of five caffeine N-demethylation genes (*ndmA*, *ndmB*, *ndmC*, *ndmD*, *ndmE*), with *Pseudomonas* spp. S31, S32, S37, S60 and *P*. *parafulva* having the full complement of these genes. Some of the bacteria carrying the ndm genes were detected in eggs, suggesting possible vertical transmission, while presence of caffeine-degrading bacteria in frass, e.g., *P. parafulva* (*ndmABCDE*) and *Bacillus aryabhattai* (*ndmA*) could result in horizontal transmission to all insect life stages. Thirty-five bacterial species associated with the insect (*Acinetobacter* sp. S40, S54, S55, *B. aryabhattai*, *B. cereus* group, *Bacillus* sp. S29, S70, S71, S72, S73, *D. lacustris*, *Erwinia* sp. S38, S43, S59, S63, *K. oxytoca*, *Kosakonia cowanii*, *Ochrobactrum* sp. S45, S46, *Paenibacillus* sp. S28, *Pantoea* sp. S61, S62, *P. aeruginosa*, *P. parafulva*, *Pseudomonas* sp. S30, S31, S32, S37, S44, S60, S75, *Stenotrophomonas* sp. S39, S41, S48, S49) might contribute to caffeine breakdown using the C-8 oxidation pathway, based on presence of genes required for this pathway. It is possible that caffeine-degrading bacteria associated with the coffee berry borer originated as epiphytes and endophytes in the coffee plant microbiota.

## Introduction

Approximately 80 plant species synthesize purine alkaloids (Ashihara and Crozier, 1999). Among these species, coffee, tea (*Camellia* spp.), and cacao (*Theobroma cacao*) are well known for the presence of the purine alkaloid 1,3,7-trimethylxanthine, commonly known as caffeine, which is widely used for its stimulant effects (Cappelletti et al., 2015). Of 125 species in the genus *Coffea* (Rubiaceae) (Davis et al., 2006, 2011), only three are commercially traded: *Coffea arabica*, *C. canephora* (also known as robusta coffee), and *C. liberica*. All three species contain different levels of caffeine, with mean levels in *C. canephora* being the highest (2.4%), followed by. *C. liberica* (1.9%), and *C. arabica* (1.3%) (Ashihara et al., 2017).

Various roles have been proposed to explain the presence of caffeine in plants: (1) inhibition of competing plants through allelopathic effects (Waller et al., 1986); (2) improved pollinator fidelity, and consequently, plant reproductive success (Wright et al., 2013); and (3) deterrency of insects (Levinson, 1976; Nathanson, 1984; Kim et al., 2006) or plant pathogens (Kim and Sano, 2008; Sano et al., 2013). Indeed, several studies have confirmed a negative effect of caffeine on insects (see Vega et al., 2003 and references therein). An exception is the coffee berry borer, *Hypothenemus hampei* (Ferrari) (Coleoptera: Curculionidae: Scolytinae), which has evolved an extreme adaptation to caffeine and is the only insect that feeds solely on seeds within the coffee fruit (Vega et al., 2015).

The coffee berry borer infestation cycle begins with an inseminated female entering a coffee fruit, followed by oviposition in galleries built throughout one or both coffee seeds within the fruit. Occasionally, a fruit might be colonized by more than one female. The insect exhibits a skewed sex ratio favoring females as well as sibling mating; therefore, females emerging from the fruit are already inseminated and ready to colonize another fruit (Vega et al., 2015). The insect causes losses in yield and quality which in Brazil alone have been estimated at US$215–358 million per year (Oliveira et al., 2013).

**FIGURE 1:**
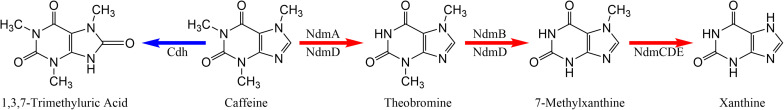
Metabolic pathways for caffeine degradation by bacteria. N-demethylation is indicated by red arrows. C-8 oxidation is indicated by the blue arrow.

Ceja-Navarro et al. (2015) demonstrated that the adaptation required by the coffee berry borer to survive on a food source containing caffeine (i.e., the coffee seed) involves a caffeine degrading mechanism provided by bacterial symbiotes (we use the term “symbiote” rather than “symbiont” based on the rationale described by Vega and Biedermann, 2020). There are two bacterial mechanisms for caffeine degradation: N-demethylation and C-8 oxidation (Summers et al., 2015). N-demethylation (Figure 1) is the most common pathway for bacterial metabolism of caffeine and requires five genes: *ndmA*, *ndmB*, *ndmC*, *ndmD*, and *ndmE*. The enzyme NdmA first removes the N_1_-methyl group from caffeine to form theobromine (3,7-dimethylxanthine), which is further converted to 7-methyxanthine by NdmB (Summers et al., 2012; Figure 1). 7-Methylxanthine then undergoes N_7_-demethylation to xanthine by the NdmCDE protein complex (Summers et al., 2013; Figure 1). Both NdmA and NdmB require the presence of NdmD for activity (Summers et al., 2012; Figure 1). NdmE is believed to serve a structural role in the NdmCDE complex and does not have a known catalytic activity (Summers et al., 2013). At least 71 bacterial strains in 27 genera and from many different habitats worldwide have been reported to be involved in caffeine degradation, with *Pseudomonas* spp. being the most common (Supplementary Table 1). Thus, caffeine degradation is a function useful in diverse ecological contexts and may have evolved several times. The observation that so many bacteria from different parts of the world (Supplementary Table 1) are able to degrade caffeine through a similar mechanism might be explained by an ancient metabolic pathway for methylxanthines or purines in general that is today retained in some bacteria or by horizontal gene transfer.

Ceja-Navarro et al. (2015) identified 14 caffeine-degrading bacteria in the alimentary canal of the coffee berry borer and only *P. fulva* tested positive for *ndmA*, implying that other caffeine demethylase genes or a different caffeine degradation mechanism (i.e., C-8 oxidation) is present in the other bacterial groups. Elimination of *P. fulva* from the coffee berry borer caused negative effects on insect fitness (Ceja-Navarro et al., 2015), thereby confirming the critical importance of the bacterial symbiosis.

For the long-term stability of a host-symbiote system, it is beneficial for both players to evolve a reliable mechanism for the transfer of symbiotes from host to host and across generations (Jaenike, 2012). Many host-symbiote systems accomplish this transfer through vertical transmission (i.e., from mother to offspring) (Ebert, 2013). For the coffee berry borer–bacteria system the mode of transmission is unknown. Acquisition of caffeine-degrading bacteria by the coffee berry borer may follow two modes: vertical transmission or horizontal transmission (i.e., among unrelated hosts, including from the environment to the host) (Jaenike, 2012; Ebert, 2013). A combination of these two modes, called mixed-mode transmission, whereby both vertical and horizontal transmission of the same symbiote occurs, may also take place and this has been reported in diverse host-symbiote systems (Ebert, 2013). To assess the mode of transmission, ideally one would study the physical course of transfer of the symbiote from host to host, i.e., the route of transmission. As the observation of the transfer itself is difficult, it is essential to first determine where the symbiotes are found in order to then make an assessment on the route of transmission. For example, if the symbiote is present inside the host’s egg, a transovum route of transmission is indicated, which is a form of vertical transmission. Presence of the symbiotes in the host’s frass allows for potential transfer of symbiotes among unrelated hosts, a form of horizontal transmission. Some routes of transmission are less clearly associated with a mode of transmission and may allow both vertical and horizontal transmission. For example, presence of symbiotes in the female host’s saliva, may allow the mother to transfer symbiotes to her offspring, but this may also happen to unrelated offspring.

Here we focus on (1) the identification of culturable bacteria associated with heads, eggs, larvae, and frass of the coffee berry borer; (2) the detection of caffeine N-demethylation or C-8 oxidation genes in these bacteria; and (3) the *in vitro* detection of caffeine metabolites for eight bacteria found to have caffeine N-demethylation genes. We also interpret these data with regard to possible modes of transmission of these bacteria. These findings are important in understanding the role of caffeine-degrading bacteria within the coffee agroecosystem, including the plant, the coffee berry borer, and the soil.

## Materials and Methods

### Insects

Coffee berry borers were dissected from infested coffee fruits collected in the field in Hawai’i and Mexico and from a colony continuously reared for ca. 15 years on an artificial diet at the United States Department of Agriculture – Agricultural Research Service laboratory in Beltsville, Maryland. Hawaiian specimens were dissected from infested *C. arabica* berries collected at: (1) Holualoa, Hawai’i (19°38′26″ N; 155°57′19″ W; 44 meters above sea level – masl); (2) two locations in Wood Valley, K’au, Hawai’i (19°16′15″ N; 155°29′19″ W; 795 masl; and 19°15′52″ N; 155°28′00″ W; 638 masl); and (3) Dole Plantation, Waialua, Oahu (21°33′51″ N; 158°04′26″ W; 146 masl). Coffee berry borers from Mexico were dissected from infested *C. canephora* fruits collected at Finca San Antonio, Cacahoatán, Chiapas (15°00′20″ N; 92°09′12″ W; 562 masl).

### Tissue Preparation and Culture

Adult female coffee berry borers (ca. 2 mm long) were sterilized by sonicating in 90% ethanol for 30 s in sterile 1.5 ml Eppendorf tubes placed in a floating foam tube rack placed inside a Branson 2210 ultrasonic cleaner (Branson Ultrasonics Corp., Danbury, CT, United States), followed by soaking in 3.5% sodium hypochlorite for 5 min (no sonication). Insects were then washed twice in nuclease-free water and blotted on a sterile paper towel in the laminar flow hood. Twenty heads from each sampling site (Table 1) were dissected from surface-sterilized adult females and macerated with sterile disposable plastic mini-pestles in 2 ml nuclease-free culture tubes containing 1 ml of nutrient broth (NB; Sigma-Aldrich, St. Louis, MO, United States). The reason for using heads is based on having observed an egg grooming behavior in which adult females smear recently oviposited eggs using her mouthparts (Vega et al., 2017), suggesting they may secrete a substance over the egg. Twenty eggs from each sampling site were cultured with or without surface-sterilization (Table 1). Eggs were sterilized for 1 min by placing in a mixture of 0.5% sodium hypochlorite and 70% ethanol. The sterilizing solution was drawn off with a pipette and replaced with nuclease-free water. Sterilized eggs were left whole or were macerated with sterile disposable plastic mini-pestles in 2 ml nuclease-free culture tubes containing 1 ml of nutrient broth (NB; Sigma-Aldrich, St. Louis, MO, United States). Whole non-sterilized eggs and female larvae (*n* = 10) from Mexico (Table 1) were directly transferred to NB (see below). Frass (Table 1) was collected with a sterilized metal spatula directly from the surface of artificial media from the Beltsville colony. The effectiveness of the sterilization protocol was assessed by placing a subsample of the nuclease-free water (used to wash the samples after removing the sterilizing solution) on the surface of nutrient agar (NA; Sigma-Aldrich, St. Louis, MO, United States) and observing whether any growth ensued, in which case the samples were discarded.

**TABLE 1:** Bacterial identification based on Illumina sequence, with GenBank *E*-values for genes involved in the caffeine N-demethylation pathway (*ndmA*, *ndmB*, *ndmC*, *ndmD*, *ndmE*), coffee berry borer source used for bacterial isolation (eggs, frass, heads, larvae), non-macerated (whole) or macerated tissue, sterile (St) or non-sterile (NSt), sampling site, GenBank accession numbers, and average nucleotide identity (ANI; Chun et al., 2018) analysis.

Isolate	Illumina Sequence ID	*ndmA*	*ndmB*	*ndmC*	*ndmD*	*ndmE*	Source	Whole/Macerated	St/NSt	Sampling Site	GenBank #	ANI
S40	*Acinetobacter* sp. S40			ndmC 2E-61	ndmD 1E-62		Eggs	Whole	St	Mexico	JACVTU000000000	84.19
S54	*Acinetobacter* sp. S54			ndmC 2E-61	ndmD 1E-62		Eggs	Whole	NSt	Oahu, HI	JACVUI000000000	84.20
S55	*Acinetobacter* sp. S55			ndmC 2E-61	ndmD 1E-62		Eggs	Whole	NSt	Oahu, HI	JACVUJ000000000	84.16
S34	*Bacillus aryabhattai*	ndmA 1E-50					Eggs	Macerated	St	Mexico	JACVTQ000000000	99.53
S35	*Bacillus aryabhattai*	ndmA 5E-51					Frass	N/A	N/A	Beltsville, MD	JACVTR000000000	95.93
S65	*Delftia lacustris*	ndmA 7E-48			ndmD 2E-45	ndmE 1E-40	Heads	Macerated	St	K’au, HI	JACVUR000000000	95.08
S66	*Delftia lacustris*	ndmA 7E-48			ndmD 2E-45	ndmE 1E-40	Heads	Macerated	St	K’au, HI	JACVUS000000000	95.10
S67	*Delftia lacustris*	ndmA 7E-48			ndmD 2E-45	ndmE 1E-40	Heads	Macerated	St	K’au, HI	JACVUT000000000	94.97
S38	*Erwinia* sp. S38	ndmA 1E-42			ndmD 6E-57		Eggs	Whole	St	Mexico	JACVTS000000000	79.12
S43	*Erwinia* sp. S43	ndmA 1E-42			ndmD 2E-59		Larvae	Whole	NSt	Mexico	JACVTX000000000	79.07
S63	*Erwinia* sp. S63				ndmD 1E-57		Heads	Macerated	St	K’au, HI	JACVUP000000000	75.50
S69	*Klebsiella oxytoca*	ndmA 1E-46			ndmD 1E-53		Heads	Macerated	St	Mexico	JACVUU000000000	99.10
S45	*Ochrobactrum* sp. S45					ndmE 5E-43	Eggs	Macerated	St	Mexico	JACVTZ000000000	83.82
S46	*Ochrobactrum* sp. S46					ndmE 5E-43	Eggs	Macerated	St	Mexico	JACVUA000000000	83.99
S61	*Pantoea* sp. S61					ndmE 5E-40	Heads	Macerated	St	Holualoa, HI	JACVUO000000000	86.23
S68	*Pseudomonas aeruginosa*				ndmD 4E-69	ndmE 6E-52	Heads	Macerated	St	Mexico	JACVZF000000000	99.42
S33	*Pseudomonas aeruginosa*				ndmD 4E-69	ndmE 6E-52	Eggs	Macerated	St	Mexico	JACVTP000000000	99.44
S36	*Pseudomonas parafulva*	ndmA E0	ndmB E0	ndmC E0	ndmD E0	ndmE 7E-155	Frass	N/A	N/A	Beltsville, MD	JACVVG000000000	98.05
S30	*Pseudomonas* sp. S30					ndmE 8E-45	Heads	Macerated	St	Beltsville, MD	JACVTO000000000	82.20
S31	*Pseudomonas* sp. S31	ndmA E0	ndmB E0	ndmC E0	ndmD E0	ndmE 4E-154	Eggs	Whole	NSt	Mexico	JACVVE000000000	86.15
S32	*Pseudomonas* sp. S32	ndmA E0	ndmB E0	ndmC E0	ndmD E0	ndmE 7E-155	Eggs	Whole	St	Mexico	JACVVF000000000	84.66
S37	*Pseudomonas* sp. S37	ndmA E0	ndmB E0	ndmC E0	ndmD E0	ndmE 7E-155	Eggs	Whole	St	Mexico	JACVZC000000000	89.53
S44	*Pseudomonas* sp. S44				ndmD 2E-65	ndmE 2E-42	Eggs	Whole	NSt	Mexico	JACVTY000000000	83.88
S60	*Pseudomonas* sp. S60	ndmA E0	ndmB E0	ndmC E0	ndmD E0	ndmE 7E-155	Heads	Macerated	St	Holualoa, HI	JACVVH000000000	84.78
S75	*Pseudomonas* sp. S75					ndmE 8E-45	Heads	Macerated	St	Beltsville, MD	JACVVA000000000	82.14

*All insects were field collected, except “Beltsville, MD”, which were reared in the laboratory (see column labeled “Sampling Site”).*

Samples (macerated sterile heads, whole sterile or non-sterile eggs, macerated sterile eggs, whole non-sterile larvae, and frass) were transferred to 4.5 ml sterile, nuclease-free culture tubes containing 2 ml NB and incubated at 28°C and 225 rpm on a rotary shaker for 48 h. The metagenomic (mixed taxa) NB cultures were archived at −80°C by combining 500 μl of each culture with 125 μl of 80% glycerol in nuclease-free deep-well plates. Ten microliters of each original culture were diluted in 50 μl of molecular-grade water (nuclease-free water) for polymerase chain reaction (PCR) screening analysis. The remainder of the 48 h NB cultures were used for dilution plating on NA (100 μl of each dilution on NA; 24 h at 28°C), to obtain single colony isolates. A total of 938 isolates were obtained: 378 from Mexico; 284 from the Beltsville colony; and 276 from Hawai’i. Single colony isolates were assigned a general morphotype based on macroscopic colony features, picked with a sterile toothpick, transferred to nuclease-free 96-well, deep-well plates containing 500 μl NB and incubated at 28°C and 225 rpm on a rotary shaker for 24 h. NB cultures of the single colony isolates were archived at −80°C (as described previously) in the Sustainable Agricultural Systems Laboratory Collection (USDA, ARS, Beltsville, MD, United States). Ten microliters of each original culture were diluted in 50 μl of nuclease-free water for PCR screening analysis.

### PCR Screening of Single Colony Isolates for 16S rRNA and ndmA Genes

Single colony NB dilutions were used as template for PCR amplification of the16S rRNA gene and of the methylxanthine N1-demethylase gene, *ndmA*. Primers used for 16S rRNA amplification were 27F (5′- AGA GTT TGA TCM TGG CTC AG-3′) and 1392R (5′- ACG GGC GGT GTG TAC A-3′), and for *ndmA* were CBBcdmF (5′- TGG CAT CCC GTW TGT ACY GT-3′) and CBBcdmR (5′- CTT GKA TAA CRA TTC GCA ACC-3′) (Ceja-Navarro et al., 2015). Twenty-five microliter PCR reactions contained 3 μl of diluted NB isolate culture, 0.4 μM of each primer and 1X AmpliTaq Gold PCR Master Mix (6.25 U AmpliTaq Gold DNA polymerase, 2.5 mM MgCl_2_, and 200 μM of each dNTP; Applied Biosystems/Life Technologies, Carlsbad, CA, United States). PCR amplification was conducted in an Eppendorf Mastercycler Gradient thermal cycler (Eppendorf, Westbury, NY, United States). The amplification program consisted of an initial 5 min denaturation step at 95° C, followed by 35 cycles at 95°C for 30 s, 54°C for 30 s, and 72°C for 1 min; and a final extension step at 72°C for 10 min. Following amplification, PCR products were electrophoresed on 1% agarose gels containing 1X TAE and 1X GelStar Nucleic Acid Gel Stain (Lonza Rockland Inc., Rockland, ME, United States), and documented using UV transillumination to detect 16S rRNA and *ndmA* bands.

### Dye-Terminator Sequencing of 16S rRNA and ndmA Products

Polymerase chain reaction products from the 16S rRNA and *ndmA* amplifications were diluted 1:3 with nuclease-free water and direct-sequenced using the BigDye Terminator v3.1 Cycle Sequencing Kit according to the manufacturer’s protocol (Applied Biosystems/Life Technologies, Carlsbad, CA, United States). Separate sequencing reactions were run for the respective forward and reverse primers in each set (27F and 1392R, CBBcdmF, and CBBcdmR). Sequencing reactions were purified using the Performa DTR Ultra 96-Well Plate Kit (EdgeBio, Gaithersburg, MD, United States), resuspended in HiDi Formamide (Applied Biosystems/Life Technologies, Carlsbad, CA, United States) and analyzed on an ABI Prism 3130 Genetic Analyzer (Applied Biosystems Inc., Foster City, CA, United States). Sequences were quality checked and aligned using the DNAStar suite of software (DNAStar, Madison, WI, United States), and preliminary identification made using the Basic Local Alignment Search Tool (BLAST) and GenBank nucleotide data bank from the National Center for Biotechnology Information, Bethesda, MD, United States^1^.

### High Throughput Sequencing

Fifty representative single colony isolates were selected for Illumina genomic library preparation using a ranking based on at least one of the following: (1) preliminary 16S rRNA BLAST identification; (2) surface-sterilization or no surface-sterilization; (3) type of insect tissue cultured; (4) sampling site; (5) colony morphotype; (6) whole or macerated tissue; (7) presence of ndmA amplicon; or (8) BLAST identification of ndmA sequence. It is important to note that ndmA was not present in all the isolates when this selection was made, as our interest was focused at casting a wide net representative of as many characteristics as possible. Single colony isolates from glycerol stocks were grown overnight in NB at 28°C and 225 rpm. Cells were pelleted at 10,000 relative centrifugal force for 1 min, and genomic DNA extracted using the Qiagen DNeasy DNA extraction protocol for bacterial cultures (Qiagen, Germantown, MD, United States). Genomic DNA quality was assessed by nanodrop 260/280 ratio, and quantity was measured using a Qubit fluorometer (Thermo Fisher Scientific Inc., Waltham, MA, United States). Libraries were prepared from 1 ng of genomic DNA from 50 coffee berry borer-associated single colony isolates using the Nextera XT DNA Library Prep Kit (Illumina, Inc., San Diego, CA, United States). The libraries were sequenced on a NextSeq by bi-directional multiplex-indexed Illumina sequencing (Nextera XT Index Kit, Illumina, Inc., San Diego, CA, United States) using a NextSeq 500 High Output Kit (150 cycles; Illumina, Inc., San Diego, CA, United States).

### Bioinformatics

The quality of the reads was checked by the FASTQC program^2^. The 50 isolates (4 paired-end libraries for each isolate) were assembled using the SPAdes assembler v3.14.0 (-t 60, -m 500, –careful, -k 17,21,31,41,51) (Bankevich et al., 2012). The read coverage (read mapping back to the contigs) was calculated using Bowtie2 (Langmead and Salzberg, 2012) and the BBtools suite^3^. Gene prediction on the contigs was performed using the software program Prodigal v2.6.3 (Hyatt et al., 2010). The gene-encoded proteins were annotated by blasting against the refseq of bacterial proteins at the NCBI using the BLASTP program. Gene-encoded proteins were also blasted against known caffeine break-down genes: KAF4561170_ndmA, KAF4561167 ndmB, KAF4561164_ndmC, KAF4561163_ndmD, KAF4561162_ndmE, KAF4561168_cafR, KAF4561173_cafT, KAF4561166_cafP, KAF4561172 frmA, KAF4561161_frmB, KAF4561171_VOC where only high *E*-values close to E 0.0 were considered to be significant matches. 16S rRNAs were predicted using RNAmmer 1.2 and tRNAs were predicted by tRNAscan-1.4. Bacterial identification was based on the top BLAST hits with the 16S rRNA followed by an analysis of the average nucleotide identity (ANI; Chun et al., 2018; Table 1) between the draft genome generated in this study with the genome of the associated top BLAST. Values > 95% were used as the species boundary following proposed minimal standards by Chun et al. (2018). The Orthologous Average Nucleotide Identity Tool (OrthoANI; Lee et al., 2016; Chun et al., 2018) software was downloaded from^4^.

### Analysis of Cell Metabolism

The 50 bacterial strains sequenced in this study were inoculated from frozen glycerol stocks into a modified M9 minimal medium containing, per liter, 12.8 g Na_2_HPO_4_⋅7H_2_O, 3 g K_2_HPO_4_, 2.76 g NH_4_Cl, 0.5 g NaCl, 493 mg MgSO_4_, 55.5 mg CaCl_2_, and 2.5 g caffeine (J. T. Baker, Avantor Performance Materials, Inc., Center Valley, PA, United States) or in nutrient broth (6 g/L peptone and 3 g/L yeast extract; VWR International LLC, Radnor, PA, United States) containing 2.5 g/L caffeine. Aliquots were removed periodically from the cultures and mixed 1:1 (vol:vol) with methanol to stop cell growth and metabolism. Concentrations of caffeine and metabolites were then determined by HPLC as described previously (Summers et al., 2012) using a Shimadzu Prominence series HPLC equipped with a photodiode array. Metabolites were identified based on their retention times and UV spectra when compared with authentic standards.

## Results

### Bacterial Associates

Assembly statistics for 50 bacterial strains in 14 genera (*Acinetobacter*, *Bacillus*, *Delftia*, *Enterococcus*, *Erwinia*, *Klebsiella*, *Kosakonia*, *Lactococcus*, *Leuconostoc*, *Ochrobactrum*, *Paenibacillus*, *Pantoea*, *Pseudomonas*, and *Stenotrophomonas*) isolated from coffee berry borer eggs, frass, head, and larvae are presented in Supplementary Table 2. Based on bioinformatics analysis, 21 bacterial species (*Acinetobacter* sp. S40, S54, S55, *Bacillus aryabhattai*, *Delftia lacustris*, *Erwinia* sp. S38, S43, S63, *Klebsiella oxytoca*, *Ochrobactrum* sp. S45, S46, *Pantoea* sp. S61, *Pseudomonas aeruginosa*, *P. parafulva*, and *Pseudomonas* sp. S30, S31, S32, S37, S44, S60, S75) contain genes homologous to those involved in caffeine N-demethylation (Table 1); 35 bacterial species (*Acinetobacter* sp. S40, S54, S55, *B. aryabhattai*, *B. cereus* group; *Bacillus* sp. S29, S70, S71, S72, S73, *D. lacustris*, *Erwinia* sp. S38, S43, S59, S63, *K. oxytoca*, *Kosakonia cowanii*, *Ochrobactrum* sp. S45, S46, *Paenibacillus* sp. S28, *Pantoea* sp. S61, S62, *P. aeruginosa*, *P. parafulva*, *Pseudomonas* sp. S30, S31, S32, S37, S44, S60, S75, *Stenotrophomonas* sp. S39, S41, S48, S49) contain genes that may be involved in C-8 oxidation (Supplementary Table 3); and nine bacterial species (*Bacillus cereus* group, *Enterococcus* sp. S52, S53, S76, S77, *Lactococcus* sp. S47, S64, and *Leuconostoc* sp. S50, S51) did not have any genes known for caffeine breakdown function (Supplementary Table 3).

### Caffeine Demethylation Genes

**FIGURE 2:**
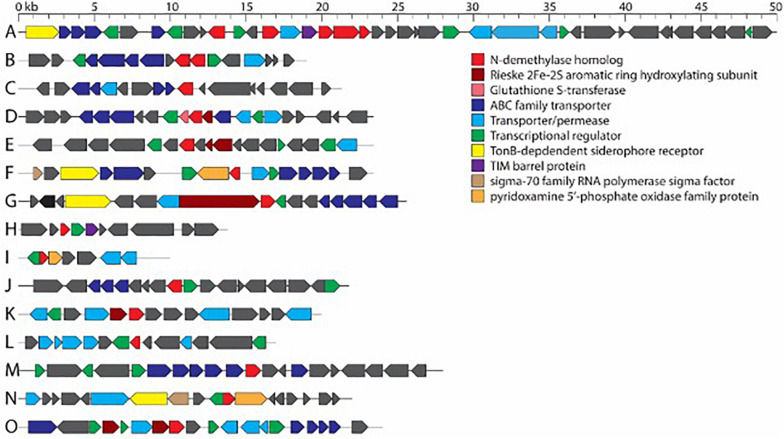
Consensus gene clusters containing *N*-demethylase genes from bacterial isolates in this work. **(A)** *ndmABCDE* cluster from *Pseudomonas* sp. S31, S32, S37, S60, and *Pseudomonas parafulva*. **(B)** *ndmAD* cluster from *Erwinia* sp. S38, S43. **(C)** *ndmA* cluster from *Bacillus aryabhattai*. **(D)** *ndmC* gene cluster from *Acinetobacter* sp. S40, S54, S55. **(E)** *ndmD* gene cluster from *Acinetobacter* sp. S40, S54, S55. **(F)** *ndmE* gene cluster from *Ochrobactrum* sp. S45, S46. **(G)** *ndmD* gene cluster from *Pseudomonas aeruginosa*. **(H)** *ndmE* gene cluster from *Pseudomonas aeruginosa*. **(I)** *ndmE* gene cluster from *Pseudomonas* sp. S30, S75. **(J)** *ndmD* gene cluster from *Erwinia* sp. S63. **(K)** *ndmD* gene cluster from *Pseudomonas* sp. S44. **(L)** *ndmE* gene cluster from *Pseudomonas* sp. S44. **(M)** *ndmA* gene cluster from *Delftia lacustris*. **(N)** *ndmD* gene cluster from *D. lacustris*. **(O)** *ndmE* gene cluster from *D. lacustris*. Genes in each cluster are listed in Supplementary Table 4.

The full complement of caffeine N-demethylation genes (*ndmABCDE*) was detected in (1) *P. parafulva* isolated from coffee berry borer frass from Beltsville; (2) *Pseudomonas* sp. S60 isolated from macerated sterilized heads from Hawai’i; (3) *Pseudomonas* sp. S31 isolated from whole non-sterilized coffee berry borer eggs from Mexico; and (4) *Pseudomonas* sp. S32, S37 isolated from whole sterilized eggs from Mexico (Table 1). In each case, the *ndmABCDE* genes were found in a gene cluster (Figure 2), with gene arrangement identical to the alkylxanthine (Alx) gene cluster encoding N-demethylase enzymes and which has been reported in *Pseudomonas* sp. CES (Summers et al., 2020) and *Pseudomonas* sp. NCIM 5235 (Retnadhas and Gummadi, 2018). A summary for the specific insect tissue from which the 21 bacterial species involved in caffeine N-demethylation were isolated is presented in Table 2. Nineteen bacterial species were isolated from only one source, i.e., heads, eggs, larvae, or frass (Table 2). Two bacterial species were isolated from two different sources: *B. aryabhattai* (eggs and frass), and *P. aeruginosa* (heads and eggs) (Table 2).

**TABLE 2:** Coffee berry borer source (heads, eggs, larvae, frass) in which bacteria with caffeine N-demethylation genes (*ndmA*, *ndmB*, *ndmC*, *ndmD*, *ndmE*) were detected.

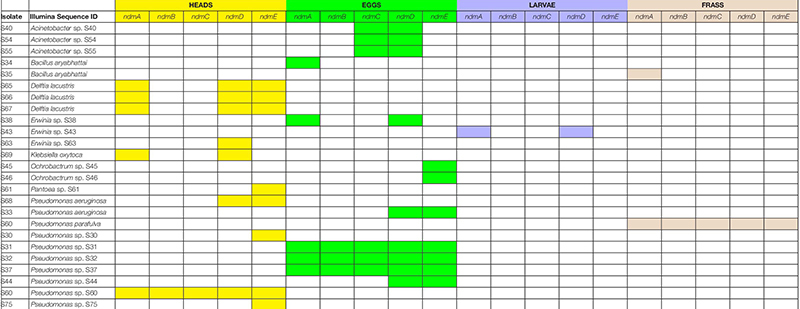

*Colored boxes across bacterial species indicate which genes were detected. Refer to Table 1 for more details on sampling sites, etc.*

**FIGURE 3:**
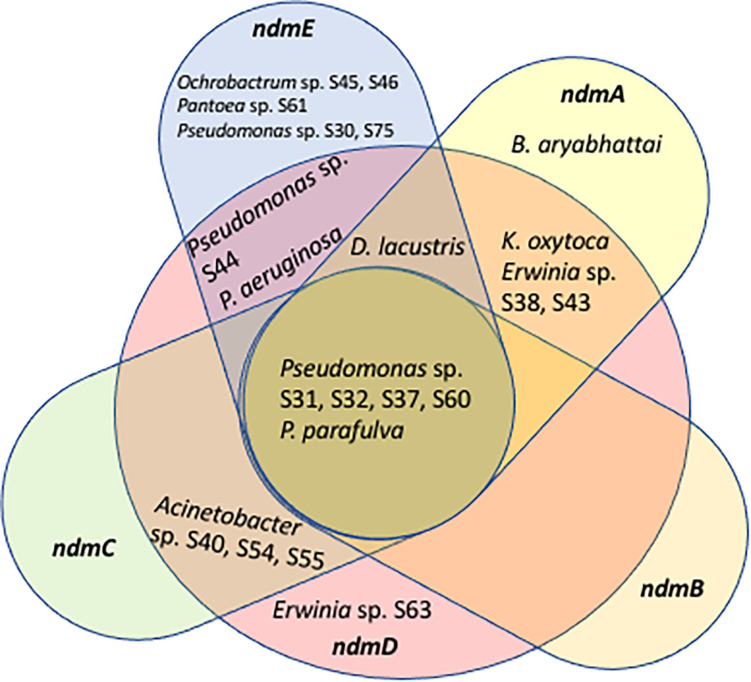
Venn diagram showing genes involved in the caffeine N-demethylation pathway shared among *Acinetobacter* sp. S40, S54, S55, *Bacillus aryabhattai*, *Delftia lacustris*, *Erwinia* sp. S38, S43, S63, *Klebsiella oxytoca*, *Ochrobactrum* sp. S45, S46, *Pantoea* sp. S61, *Pseudomonas* sp. S30, S31, S32, S37, S44, S60, S75, *P. aeruginosa*, and *P. parafulva*.

Some of the ndm genes were also detected in *Acinetobacter* sp. S40, S54, S55 (*ndmCD*), *B. aryabhattai* (*ndmA*), *D. lacustris* (*ndmADE*), *Erwinia* sp. S38, S43 (*ndmAD*), *Erwinia* sp. S63 (*ndmD*), *K. oxytoca* (*ndmAD*), *Ochrobactrum* sp. S45, S46 (*ndmE*), *Pantoea* sp. S61 (*ndmE*), *P. aeruginosa* (*ndmDE*), *Pseudomonas* sp. S30, S75 (*ndmE*), and *Pseudomonas* sp. S44 (*ndmDE*) (Table 1). A Venn diagram showing how the genes involved in the caffeine N-demethylation pathway are shared among the various bacterial species is shown in Figure 3.

Aside from *ndmAD* in *Erwinia* sp. S38, S43, which were found adjacent to each other (Figure 2), no other *ndm* gene homologs were in the same gene cluster. Rather, these genes were scattered throughout the genome (Figure 2). However, most of the homologs were surrounded by similar genes such as ABC family and other transporters, permeases, and GntR, LysR, and MarR transcriptional regulators (Figure 2; Supplementary Table 4).

While Pseudomonads are common caffeine degraders, two *Acinetobacter* sp. strains, a *Klebsiella*/*Rhodococcus* mixture, three *Pantoea* species, an *Ochrobactrum* sp., and *S. maltophilia* have been previously reported to degrade caffeine (Supplementary Table 1). Thus, the diversity observed here is not surprising but adds additional genera, such as *Bacillus*, *Delftia*, and *Erwinia* to the list of potential caffeine-degrading bacteria.

### Analysis of Cell Metabolism

Of the 50 strains tested grown in M9 minimal media containing 0.25% caffeine as the sole carbon source to detect metabolites, only eight grew and produced sufficient metabolites to facilitate their detection by HPLC (Table 3). In each case, we detected theobromine and 7-methylxanthine in the growth medium, indicating that caffeine is metabolized by *N*-demethylation. The remaining 42 strains did not grow when caffeine was provided as the sole carbon and nitrogen source.

**TABLE 3:** Strains with caffeine N-demethylation genes and/or metabolites isolated from coffee berry borers, coffee plantation soils, and coffee plants.

Coffee berry borer (present paper)	Coffee berry borer (Ceja-Navarro et al., 2015)	Coffee plantation soils	Coffee plants
*Pseudomonas aeruginosa* **(S68)**	*Brachybacterium rhamnosum*	*Acinetobacter* sp. (Yamaoka-Yano and Mazzafera, 1998)	*Pseudomonas* sp. (Baker et al., 2012)
***Pseudomonas parafulva***	*Enterobacter sp.*	Coryneform bacterium (Yamaoka-Yano and Mazzafera, 1998)	*P. monteilii* (Arimurti et al., 2018)
*Pseudomonas* sp. S30, S31, **S32**, **S37**, S44, **S60**, S75	*Jonesiae*	*Flavobacterium* sp. (Yamaoka-Yano and Mazzafera, 1998)	*P. putida* (Nunes and de Melo, 2006)
*Acinetobacter* sp. S40*, S54*, S55*	*Kosakonia cowanii*	*Pseudomonas* sp.** (Dash and Gummadi, 2006)	
Bacillus aryabhattai*	*Microbacterium binotii*	*Pseudomonas* sp. (Gokulakrishnan et al., 2007)	
Delftia lacustris*	*Novosphigobium* sp.	*Pseudomonas alcaligenes* (Sarath Babu et al., 2005)	
*Erwinia* sp. S38*, S43*, S63*	*Ochrobactrum sp.*	*Pseudomonas putida* (Yamaoka-Yano and Mazzafera, 1998)	
Klebsiella oxytoca*	*Pantoea eucalypti*	Pseudomonas putida** (Yamaoka-Yano and Mazzafera, 1999)	
*Ochrobactrum* sp. S45*, S46*	*Pantoea septica*	Serratia marcescens** (Mazzafera et al., 1996)	
*Pantoea* sp. S61*, **S62**	*Pantoea vagans*		
*Bacillus* sp. **S72**,	*Pseudomonas sp.*		
*Stenotrophomonas* sp. **S39**	*Pseudomonas fluorescens*		
	*Pseudomonas fulva*		
	*Stenotrophomonas maltophilia*		

*In column one, *Pseudomonas* sp. S32, S37, S60 and *P. parafulva*, had the full complement of N-demethylation genes (*ndmABCDE*) and caffeine breakdown metabolites were detected by HPLC; *Pseudomonas* sp. S31 had the full complement of N-demethylation genes but metabolites were not detected; an asterisk denotes that one, two, or three N-demethylation genes were identified, but no N-demethylation metabolites were detected, i.e., N-demethylation is not being expressed; no N-demethylation genes were detected in *Bacillus* sp. S72, *Pantoea* sp. S62, and *Stenotrophomonas* sp. S39, but metabolites were detected; *ndmD* and *ndmE*, as well as N-demethylation metabolites, were detected in *Pseudomonas aeruginosa*. Of the 50 strains isolated from the coffee berry borer, eight listed in bold in the first column were the only ones capable of growing in M9 minimal media containing 0.25% caffeine as the sole carbon source. Among all the bacteria listed in the second column, N-demethylation was only positively determined for *P. fulva* (Ceja-Navarro et al., 2015). Two asterisks in the third column indicates N-demethylation; otherwise, the caffeine breakdown mechanism was not determined. The caffeine breakdown mechanism was not determined for bacteria isolated from coffee plants (fourth column).*

## Discussion

### Distribution of Caffeine-Degrading Genes

The presence of the full complement of *ndm* genes in several Pseudomonads was expected, given that N-demethylation is the most commonly observed caffeine degradation mechanism in bacteria (Supplementary Table 1) and that *Pseudomonas* is the most common caffeine-degrading genus worldwide (Supplementary Table 1). In contrast to these strains, only a subset of the genes was found in several bacterial strains. It is possible that the bacterial cells use other undiscovered genes and enzymes for caffeine metabolism or that the similarity of the homologous genes is lower than our cutoff threshold. To minimize false positives, E-values close to E 0.0 with high sequence similarity to known ndm genes were considered, since ndm genes have sequence similarity to proteins that might not be involved in caffeine breakdown. Similarly, the portion of the genome containing the relevant ndm and caffeine breakdown genes in these strains may not have been sequenced due to what Baptista and Kissinger (2019) refer to as “troublesome characteristics”, including extreme nucleotide bias, long homopolymeric runs, repetitive sequences, mobile elements, etc.

Another possibility for the low number of strains with a full complement of *ndm* genes (Figure 3) is that some strains may be able to carry out one step of the caffeine degradation pathway and rely on other strains to carry out additional steps. For example, the *D. lacustris*, *Erwinia* sp. S38, S43, and *K. oxytoca* strains contain *ndmAD* homologs, which may enable them to convert caffeine to the less-toxic theobromine. *Acinetobacter* sp. S40, S54, S55 contains *ndmCD* genes, which could potentially convert 7-methylxanthine to xanthine, which is formed during the biosynthesis of caffeine in coffee plants (Ashihara et al., 1996). Therefore, drivers for bacteria to carry out individual steps in the process may be to reduce toxicity of caffeine or to use metabolites already present, such as theobromine or 7-methylxanthine. Curiously, none of the strains with only a subset of genes contain *ndmB* homologs. However, given that as few as two mutations to the *ndmA* active site are enough to change the activity from N_1_- to N_3_-demethylation (Kim et al., 2019) some of the genes identified as *ndmA* may have a broader positional specificity than those which have already been characterized. Alternatively, there may be unculturable bacteria with *ndmB* homologs that we were not able to detect using the approach described here. NdmE is a glutathione-*S*-transferase homolog and is believed to play a structural role in the NdmCDE complex; there is no known catalytic role for NdmE (Summers et al., 2013). Thus, strains that only contain *ndmE*, such as *Ochrobactrum* sp. S45, S46 and *Pantoea* sp. S61, may not have any N-demethylation activity but may have a *ndmE* homolog that carries out a different function in the cell.

We note that the presence of ndm gene homologs does not indicate that the strains can completely degrade caffeine, or even that those genes are involved in caffeine metabolism. However, there are no other reports of *N*-demethylase enzymes capable of degrading caffeine and the NdmABCDE enzymes have only exhibited activity toward caffeine and related methylxanthines. These enzymes are part of the Rieske non-heme iron oxygenase family, which contains enzymes responsible for degradation of aromatic compounds toluene, naphthalene, biphenyl, and the herbicide dicamba (Gibson and Parales, 2000). Thus, there may be other aromatic compounds toward which these gene products are active. Regardless, the presence of *ndm* gene homologs does give insight into possible mechanisms and provides the basis for further studies to elucidate caffeine degradation by bacteria.

In each of the eight strains grown in M9 minimal media with 0.25% caffeine as the sole carbon and nitrogen source (Table 3) and which produced sufficient metabolites for HPLC detection, we detected the production of theobromine and 7-methylxanthine, indicating an active N-demethylation pathway. The *P. parafulva* and *Pseudomonas* sp. S32, S37, S60 containing the full set of ndm genes were found among this group. The other four strains that grew were *Bacillus* sp. S72, *Pantoea* sp. S62, and *Stenotrophomonas* sp. S39 among which no ndm genes were detected, and *P. aeruginosa*, in which only *ndmD* and *ndmE* were present. The ability of these strains to carry out *N*-demethylation of caffeine indicates that they may possess different genes encoding enzymes of similar function, the ndm genes were simply incompletely sequenced or not sequenced (Baptista and Kissinger, 2019), or that the ndm gene E-values did not meet our cutoff threshold. To date, no other genes/enzymes have been shown to carry out *N*-demethylation of caffeine or other methylxanthines in bacteria.

The 42 strains that did not grow when caffeine was provided as the sole carbon and nitrogen source may not have all of the genes necessary to completely degrade caffeine. Another explanation is that the genes were not induced under the growth conditions. It may be possible for strains with *ndmAD* gene combinations to convert caffeine to theobromine, but the single methyl group removed would likely not enable cells to grow. Additionally, very little is known about the regulation of the caffeine-degrading genes. Studies have indicated that these genes are regulated very tightly and only expressed under certain conditions. For example, *P. putida* CBB5 can express the ndm genes when grown with 0.25% caffeine and 0.4% soytone, but expression is greatly reduced when soytone is replaced with yeast nitrogen base (YNB) (Yu et al., 2009). In contrast, the caffeine dehydrogenase expression in *Pseudomonas* sp. CBB1 is not inhibited by YNB but is almost completely repressed by soytone (Yu et al., 2008). Thus, the absence of metabolites does not indicate a lack of genes, but observation of caffeine metabolites does provide evidence for specific enzymes. Indeed, we did not observe any caffeine degradation from any of the 50 strains when grown in nutrient broth supplemented with 0.25% caffeine, indicating that there are specific circumstances in which the *ndm* genes are expressed.

We also analyzed caffeine dehydrogenase genes (*cdhA*, *cdhB*, *cdhC*) involved in C-8 oxidation, the second pathway of caffeine break down (Figure 1; Supplementary Table 3). The C-8 oxidation pathway involves the oxidation of caffeine to 1,3,7-trimethyluric acid (TMU; Figure 1; Supplementary Table 3), which is further degraded in a pathway homologous to uric acid metabolism. The caffeine dehydrogenase enzyme, encoded by genes *cdhABC*, carries out the first step in this pathway (Yu et al., 2008). Further steps are catalyzed by trimethyluric acid-degrading genes (*tmuM*, *tmuH*, *tmuD*) (Mohanty et al., 2012). The peptide sequence of the *cdhA* gene, which forms one subunit of the heterotrimeric caffeine dehydrogenase is similar to xanthine dehydrogenases, aldehyde dehydrogenases, and carbon monoxide dehydrogenases (Yu et al., 2008). Similarly, the *tmuM*, *tmuH*, and *tmuD* genes have homologs involved in uric acid metabolism. It is possible that the genes listed in Supplementary Table 3 are instead involved in xanthine and uric acid metabolism. However, xanthine dehydrogenase is not known to metabolize caffeine, nor have the *tmu* genes shown activity toward trimethyluric acid. To date, we do not have experimental evidence of TMU formation from caffeine by any of these strains, indicating that either the homologs we detected do not metabolize caffeine or that the conditions for their expression have not yet been identified. Cloning and expression of the genes and characterization of the enzymes they encode would be necessary to determine if these genes are specific for caffeine.

For strains such as *Ochrobactrum* sp. S45, S46, *Pantoea* sp. S61, and *Pseudomonas* sp. S30, S75 that have *ndmE* and *cdh* homologs, it is more likely that they metabolize caffeine through the C-8 oxidative route rather than the N-demethylation route because NdmE has no known catalytic activity. Two *B. aryabhattai* strains contain the full set of C-8 oxidative genes, but only the *ndmA* gene from the N-demethylation pathway. Because this strain does not contain an *ndmD* homolog necessary for N-demethylation, it would most likely use the oxidative route, as well.

Possible involvement of the *cdh* and *tmu* genes (Supplementary Table 3) in the breakdown of caffeine and subsequent products needs to be experimentally determined to ascertain whether this role could be occurring in the coffee berry borer. It is interesting that ndm genes were not identified in *K. cowanii*, although it has *CdhABC* and *TmuMD*; Ceja-Navarro et al. (2015) identified *K. cowanii* as a caffeine-degrading bacterium in the coffee berry borer.

It is also important to note that there might be other unculturable bacteria that are capable of degrading caffeine, although that was not the focus of this paper.

### *Pseudomonas* and *Delftia* in the Coffee Berry Borer

The genus *Pseudomonas* comprises ca. 150 species (Pereira et al., 2018) and in the present study, *Pseudomonas aeruginosa*, *P. parafulva*, and *Pseudomonas* sp. S30, S31, S32, S37, S44, S60, S75 were found to have caffeine N-demethylation genes, with five of them having the full complement of caffeine demethylating genes (*ndmABCDE*; Tables 1, 2 and Figure 3): *P*. *parafulva* (frass), *Pseudomonas* sp. S60 (heads), and *Pseudomonas* sp. S31, S32, S37 (eggs). *Pseudomonas aeruginosa* had *ndmDE* (heads, eggs) and *Pseudomonas* sp. S30, S75 only had *ndmE* (heads) (Tables 1, 2 and Figure 3).

Mariño et al. (2018) reported 392 bacterial genera in the microbiome of eggs and whole adult female coffee berry borers, with a preponderance of *Pseudomonas* and *Pantoea*. *Pseudomonas* spp. represented 48.5% of the microbiota, with *P. chloroaphis* being common in laboratory-reared insects and *P. putida* being more common in field-collected insects. Two other *Pseudomonas* species identified in the microbiome were *P. viridiflava* and *P. fulva*.

This is the first report of potential caffeine N-demethylation genes in *D. lacustris* (*ndmADE*). Intriguingly, as for *Pseudomonas* sp. S30, S60, S75, the detection was made from sterilized, macerated heads. *Delftia* has been isolated from the coffee berry borer by Mariño et al. (2018).

### Possible Modes of Bacterial Transmission

Transmission of a symbiote from the mother to its offspring is known as vertical transmission (Jaenike, 2012; Ebert, 2013; Kucuk, 2020) and could be mediated internally or externally by the ovum and referred to as transovum transmission (Solter, 2006). One type of transovum transmission is transovarial transmission involving the internal invasion of the egg by the symbiote, as has been reported for many insects (Zchori-Fein et al., 2006; Skaljac et al., 2010). We detected eight bacteria with N-demethylation genes in sterile eggs: *Acinetobacter* sp. S40 (*ndmCD*), *B*. *aryabhattai* (*ndmA*), *Erwinia* sp. S38 (*ndmAD*), *Ochrobactrum* sp. S45, S46 (*ndmE*), *P*. *aeruginosa* (*ndmDE*), and *Pseudomonas* sp. S32, S37 (*ndmABCDE*) (Table 1). It is unlikely that *Acinetobacter* sp. S40, *B*. *aryabhattai*, *Erwinia* sp. S38, and *Ochrobactrum* sp. S45, S46 are transovarially transmitted and detection might have been a result of contamination or imperfect sterilization. Transovarial transmission would be rather surprising for these multiple free-living bacteria which readily grow on standard cultivation media and are otherwise not consistently associated with the host. Using fluorescent *in situ* hybridization, Sayaka Aoki (University of Hawai’i; personal communication) has detected *Pseudomonas* spp. with at least one caffeine degrading gene inside coffee berry borer eggs.

The other type of ovum mediated transmission involves inoculation of the egg shell via frass or secretions containing the symbiote, which is ingested upon egg hatching (Hosokawa et al., 2012; Salem et al., 2015b). As stated above, we have observed an egg grooming behavior in which female coffee berry borers appear to smear her eggs shortly after oviposition (Vega et al., 2017). A similar situation has been reported in *Adomerus* stinkbugs, which smear a secretion containing a symbiote over the eggs (Hosokawa et al., 2013). The detection of *ndmABCDE* in *Pseudomonas* sp. S60 isolated from macerated sterilized heads (Table 1) could be a mechanism for vertical transmission if the hatching embryos acquire the bacterial symbiote by eating the egg shell or becoming otherwise contaminated with the symbiote. This possible type of vertical transmission needs further study.

Coffee berry borer larvae could acquire *Acinetobacter* sp. S54, S55 (*ndmCD*) and *Pseudomonas* sp. S31 (*ndmABCDE*), which were isolated from non-sterile eggs (Table 1), from the egg shell as the larvae hatches. These bacteria could also contaminate the entire environment within the coffee berry. The coffee berry borer rectum ends with the anus, which is an opening independent of the genital orifice (gonopore) (Alba-Alejandre et al., 2019). Both orifices are very close to each other, with the anal opening being above the genital orifice. Due to the large size of coffee berry borer eggs, it is very likely that as they are being oviposited they will place pressure on the rectum, which could expel fecal matter that would then contaminate the egg shell.

In addition, the presence of *ndmABCDE* in *P. parafulva* and *ndmA* in *B. aryabhattai* isolated from coffee berry borer frass from the Beltsville colony (Table 1) suggests that potential caffeine demethylating bacteria could contaminate the egg shell or be acquired from the environment (i.e., horizontal transmission) via bacterial dissemination from frass through insect contact, probing, and subsequent spread in galleries. The acquisition of bacteria through coprophagy (Salem et al., 2015a; Onchuru et al., 2018) cannot be ruled out for the coffee berry borer. *Erwinia* sp. S43 (*ndmAD*) was detected in non-sterile larvae from Mexico (Table 1), suggesting they could be acquiring the bacterium inside the gallery. Horizontal transmission of caffeine-degrading bacteria also needs further study.

### Caffeine-Degrading Bacteria in Soil and Plants

Caffeine-degrading functions might also be expressed by bacteria isolated from soil in coffee plantations, as has been shown for *Pseudomonas* sp. (Dash and Gummadi, 2006; Gokulakrishnan et al., 2007), *P. alcaligenes* (Sarath Babu et al., 2005), *P. putida* (Yamaoka-Yano and Mazzafera, 1998, 1999), and *Serratia marcescens* (Mazzafera et al., 1996; Supplementary Table 1). In addition, endophytic bacteria in coffee plants might also be capable of degrading caffeine. Vega et al. (2005) identified 19 bacterial genera as endophytes in coffee plants from Colombia, Hawai’i, and Mexico, with *Bacillus*, *Burkholderia*, *Clavibacter*, *Curtobacterium*, *Escherichia*, *Micrococcus*, *Pantoea*, *Pseudomonas*, *Serratia*, and *Stenotrophomonas* isolated from the seed. *Pseudomonas* sp., *P. chloroaphis*, and *P. putida* were identified as endophytes in various coffee plant tissues sampled (Vega et al., 2005) and *P. monteilii* has been isolated from coffee pulp waste (Arimurti et al., 2018). A coffee plant endophytic *Pseudomonas* sp. isolated in India was capable of breaking down 98.6% of the caffeine added to a nutrient broth, and six additional unidentified endophytic bacteria were also capable of catabolizing caffeine with a lower efficiency (Baker et al., 2012). Two *P. putida* strains endophytic in *C. arabica* and *Coffea canephora* leaves, stems, and roots in Brazil were capable of degrading caffeine (Nunes and de Melo, 2006).

For the coffee berry borer, the presence of bacteria on the surface of the fruit, or endophytically in the pulp or seed (Vega et al., 2005), might result in the acquisition of caffeine-degrading bacteria as the colonizing female infests fruit and seeds. A similar situation has been reported for 13 bark beetle species in the genus *Dendroctonus*, which acquire bacterial members of their microbiome from the environment, including their host trees (Hernández-García et al., 2017).

## Conclusion

The detection of genes involved in the two mechanisms for bacterial metabolism of caffeine, i.e., N-demethylation and C-8 oxidation, has revealed the complexity of culturable bacterial symbiotes of the coffee berry borer. The results could serve as a basis for in-depth studies aimed at determining if these bacteria become established in the alimentary canal of the insect and whether their elimination reduces insect fitness, as has been shown for *P. fulva* (Ceja-Navarro et al., 2015). In addition, the study serves as a first attempt to determine possible mechanisms for vertical and horizontal transmission of some of the genes involved in N-demethylation. The isolation and identification of culturable bacteria for an assessment of caffeine-breakdown capabilities would be useful in understanding the coffee berry borer microbial diversity in various coffee-growing regions as well as various coffee- growing regimes such as Arabica vs robusta and shaded coffee vs coffee grown at full sun.

The bacteria involved in the N-demethylation metabolic pathway for caffeine degradation in the coffee berry borer (Tables 1, 2 and Figure 3) likely originated as epiphytes and endophytes in the coffee plant, as a caffeine-degrading mechanism is necessary in order for the bacteria to thrive on coffee. Vega et al. (2005) identified 19 genera of endophytic bacteria in coffee plants from Colombia, Hawai’i, and Mexico, including genera potentially capable of breaking down caffeine, e.g., *Enterobacter*, *Klebsiella*, *Pantoea*, *Pseudomonas*, *Serratia*, and S*tenotrophomonas* (Table 3). Many of the bacteria associated with coffee plants might be coffee plant mutualists, deriving nutrition as well as a substrate from the plant and providing caffeine-degrading functions on leaves and fruits falling on the soil, and within the soil itself, thus recycling nutrients (Kennedy, 1999) and possibly lowering caffeine concentrations in the soil. Eight bacterial species isolated from soil in coffee plantations are capable of breaking down caffeine (Table 3). The bacterial composition in coffee fruits, leaves and roots needs to be identified and compared to the bacterial community in the soil, as well as in the coffee berry borer, in order to understand shared bacterial composition and functional redundancy (Kennedy, 1999; Nannipieri et al., 2017) in terms of caffeine-degrading genes.

## Data Availability Statement

The datasets presented in this study can be found in online repositories. The names of the repository/repositories and accession number(s) can be found at: https://www.ncbi.nlm.nih.gov/bioproject/PRJNA659418.

## Author Contributions

FEV, SE, AS, and JEM conceived and designed the coffee berry borer experiments. FEV, SE, and AS performed the coffee berry borer experiments. MBM and RMS conceived, designed, and performed the experiments related to caffeine metabolites. JS performed the bioinformatics analysis. FEV, SE, JS, RMS, and MBM analyzed the data. All authors interpreted the results and provided significant comments and inputs to the manuscript.

## Conflict of Interest

The authors declare that the research was conducted in the absence of any commercial or financial relationships that could be construed as a potential conflict of interest.

## Footnotes

1 http://www.ncbi.nlm.nih.gov/

2 https://www.bioinformatics.babraham.ac.uk/publications.html

3 https://jgi.doe.gov/data-and-tools/bbtools/

4 https://www.ezbiocloud.net/tools/orthoani

## Supplementary Material

Supplementary Table 1 At least 71 bacterial strains in 27 genera have been reported to be capable of caffeine breakdown, with *Pseudomonas* spp. being the most common.

Supplementary Table 2 Assembly statistics for 50 bacterial strains isolated from the coffee berry borer.

Supplementary Table 3 Bacteria isolated from the coffee berry borer, indicating which strains might have C-8 oxidation activity (*cdhA*, *cdhB*, *cdhC*, *tmuM*, *tmuH*, *tmuD*), coffee berry borer source used for bacterial isolation (eggs, frass, heads, larvae), non-macerated (whole) or macerated tissue, sterile (St) or non-sterile (NSt), sampling site, GenBank accession numbers, and average nucleotide identity (ANI; Chun et al., 2018) analysis. All insects were field collected, except “Beltsville, MD”, which were reared in the laboratory (see column labeled “Sampling Site”).

Supplementary Table 4 Arrangement of gene clusters.
