# Supplementary material for: Cultivation and Genome Sequencing of Bacteria Isolated From the Coffee Berry Borer (Hypothenemus hampei), With Emphasis on the Role of Caffeine Degradation
Source: Front Microbiol. 2021 Apr 6;12:644768. doi: 10.3389/fmicb.2021.644768 (PMC8055839; doi:10.3389/fmicb.2021.644768)
Supplement: Supplementary Table 1 — At least 71 bacterial strains in 27 genera have been reported to be capable of caffeine breakdown, with Pseudomonas spp. being the most common. [file Table_1.docx]

**Supplementary Table 1.** At least 71 bacterial strains in 27 genera have been reported to be capable of caffeine breakdown, with *Pseudomonas* spp. being the most common.

| **Bacterial id** | **Catabolic pathway** | **Specimen isolated from:** | **Reference** |
| --- | --- | --- | --- |
| *Acinetobacter* sp. (2 strains) | N.R. | soil in coffee plantations – Brazil | Yamaoka-Yano and Mazzafera 1998 |
| *Alcaligenes* sp. CF8 | C-8 oxidation | surface of lake water – Canada | Mohapatra et al. 2006 |
| *Arthrobacter arilaitensis* | N.R. | compost – New York (USA) | Divine 2014 |
| *Bacillus coagulans* | N.R. | soil – California (USA) | Kurtzman Jr. and Schwimmer 1971 |
| *Brachybacterium rhamnosum* | N.R. | insect – Hawaii (USA)/Mexico | Ceja-Navarro et al. 2015 |
| *Brevibacterium* sp. MTCC 10313 | N. R. | coffee pulp – India | Nayak et al. 2012 |
| Coryneform | N.R. | soil in coffee plantations – Brazil | Yamaoka-Yano and Mazzafera 1998 |
| *Cupriavidus* sp. D384 | N.R. | forest soil – Japan | Watahiki and Kimura 2017 |
| *Enterobacter* sp. | N.R. | insect – Hawaii (USA)/Mexico | Ceja-Navarro et al. 2015 |
| *Flavobacterium* sp. | N.R. | soil in coffee plantations – Brazil | Yamaoka-Yano and Mazzafera 1998 |
| Jonesiaceae | N.R. | insect – Hawaii (USA)/Mexico | Ceja-Navarro et al. 2015 |
| *Klebsiella* – *Rhodococcus* (mixed culture) | C-8 oxidation | caffeine-enriched soil – India | Madyastha and Sridhar 1998 |
| *Kosakonia cowanii* | N.R. | insect – Hawaii (USA)/Mexico | Ceja-Navarro et al. 2015 |
| *Leifsonia* sp. SIU | N.R. | soil – Malaysia | Ibrahim et al. 2016a, 2016b |
| *Methylorubrum populi* Pinkel | N-demethylation | compost containing coffee grounds – California (USA) | Parales et al. 2019 |
| *Microbacterium binotii* | N.R. | insect – Hawaii (USA)/Mexico | Ceja-Navarro et al. 2015 |
| *Novosphigobium* sp. | N.R. | insect – Hawaii (USA)/Mexico | Ceja-Navarro et al. 2015 |
| *Ochrobactrum sp.* | N.R. | insect – Hawaii (USA)/Mexico | Ceja-Navarro et al. 2015 |
| *Paenibacillus cookii* | N.R. | compost – New York (USA) | Divine 2014 |
| *Pantoea eucalypti* | N.R. | insect – Hawaii (USA)/Mexico | Ceja-Navarro et al. 2015 |
| *Pantoea septica* | N.R. | insect – Hawaii (USA)/Mexico | Ceja-Navarro et al. 2015 |
| *Pantoea vagans* | N.R. | insect – Hawaii (USA)/Mexico | Ceja-Navarro et al. 2015 |
| *Paraburkholderia caffeinilytica* CF1 | N-demethylation | tea plantation – China | Sun et al. 2020 |
| *Pseudomonas* sp. | N.R. | endophytic in *Coffea arabica* stems, roots, leaves – India | Baker et al. 2012 |
| *Pseudomonas* sp. No. 6 | N-demethylation | soil – Japan | Asano et al. 1993 |
| *Pseudomonas* sp. NCIM 5235 | N-demethylation | soil in coffee plantation - India | Dash and Gummadi 2006 |
| *Pseudomonas* sp. TH1 | N-demethylation | wastewater treatment plant – Canada | Topp et al. 2006 |
| *Pseudomonas* sp. GSC 1182 | N.R. | soil in coffee plantation – India | Gokulakrishnan et al. 2007 |
| *Pseudomonas* sp. CBB1 | C-8 oxidation | caffeine-enriched soil – Iowa (USA) | Yu et al. 2008 |
| *Pseudomonas sp.* | N.R. | insect – Hawaii (USA)/Mexico | Ceja-Navarro et al. 2015 |
| *Pseudomonas* sp. CES | N-demethylation | caffeine-enriched soil – Iowa (USA) | Summers et al. 2020 |
| *Pseudomonas alcaligenes* CFR 1708 | N.R. | soil coffee plantation – India | Sarath Babu et al. 2005 |
| *Pseudomonas pseudoalcaligenes* TPS8 | N.R. | soil in tea plantation – Iran | Ashengroph and Ababaf 2013 |
| *Pseudomonas aeruginosa* | uncertain^[[1]](#footnote-1)^ | N.R. | Franke and Hahn 1955 |
| *Pseudomonas fluorescens* NRRL B-8052, NRRL B-8053 | N-demethylation | N.R. | Haas and Stieglitz 1980 |
| *Pseudomonas fluorescens* | N.R. | insect – Hawaii (USA)/Mexico | Ceja-Navarro et al. 2015 |
| *Pseudomonas fragi* | N.R. | compost – New York (USA) | Divine 2014 |
| *Pseudomonas fulva* | N-demethylation | insect – Hawaii (USA)/Mexico | Ceja-Navarro et al. 2015 |
| *Pseudomonas monteilii* | N.R. | compost – New York (USA) | Divine 2014 |
| *Pseudomonas monteilii* KRM9 | N.R. | coffee pulp waste – Indonesia | Arimurti et al. 2018 |
| *Pseudomonas plecoglossicida* | N.R. | compost – New York (USA) | Divine 2014 |
| *Pseudomonas putida* 40 | N-demethylation | soil – California (USA) | Woolfolk 1975 |
| *Pseudomonas putida* C1 | N-demethylation | soil – Germany | Blecher 1976; Blecher and Lingens 1977; Hohnloser et al. 1980 |
| *Pseudomonas putida* NRRL B-8051 | N-demethylation | N.R. | Haas and Stieglitz 1980 |
| *Pseudomonas putida* C 3024 | N.R. | soil – The Netherlands | Middelhoven and Bakker 1982 |
| *Pseudomonas putida* WS | N-demethylation | soil – Germany | Glück and Lingens 1987, 1988 |
| *Pseudomonas putida* No. 352 | N-demethylation | soil – Japan | Asano et al. 1993, 1994 |
| *Pseudomonas putida* IF-3 | N-demethylation | soil – Japan | Koide et al. 1996 |
| *Pseudomonas putida* ATCC 700097 | N-demethylation | wastewater – California (USA) | Ogunseitan 1996, 2002 |
| *Pseudomonas putida* (7 strains) | N.R. | soil in coffee plantations – Brazil | Yamaoka-Yano and Mazzafera 1998 |
| *Pseudomonas putida* L | N-demethylation | soil in coffee plantation – Brazil | Yamaoka-Yano and Mazzafera 1999 |
| *Pseudomonas putida* KD6 | N-demethylation | N.R. | Sideso et al. 2001 |
| *Pseudomonas putida* O1G, 13R | N.R. | endophytic in *C. arabica* and *Coffea canephora* leaves, stems, roots – Brazil | Nunes and de Melo 2006 |
| *Pseudomonas putida* CBB5 | N-demethylation | caffeine-enriched soil – Iowa (USA) | Yu et al. 2009; Quandt et al. 2015 |
| *Pseudomonas putida* CT25 | N.R. | soil in tea plantation – China | Fan et al. 2011 |
| *Pseudomonas putida* | N.R. | compost – New York (USA) | Divine 2014 |
| *Pseudomonas rhizosphaerae* | N.R. | compost – New York (USA) | Divine 2014 |
| *Pseudomonas stutzeri* Gr21ZF | N.R. | soil – Lebanon | El-Mched et al. 2013 |
| *Rhodococcus opacus* M213 | N.R. | gasoline contaminated soil – Idaho (USA) | Pathak et al. 2013 |
| *Serratia marcescens* | N-demethylation | soil in coffee plantation – Brazil | Mazzafera et al. 1996 |
| *Sphingobacterium mizutaii* | N.R. | compost – New York (USA) | Divine 2014 |
| *Stenotrophomonas maltophilia* | N.R. | insect – Hawaii (USA)/Mexico | Ceja-Navarro et al. 2015 |
| *Streptomyces carpaticus* | N.R. | compost – New York (USA) | Divine 2014 |
| *Streptomyces sampsonii* | N.R. | compost – New York (USA) | Divine 2014 |

**References**

Arimurti, S., T. Ardyati, Y. Nurani, T. A. Siswoyo, and S. Suharjono. 2018. Degradation of caffeine by *Pseudomonas monteilii* KRM9. Malaysian J. Microbiol. 14:55–60.

Asano, Y., T. Komeda, and H. Yamada. 1993. Microbial production of theobromine from caffeine. Biosci. Biotech. Biochem. 58:1286–1289.

Asano, Y., T. Komeda, and H. Yamada. 1994. Enzymes involved in theobromine production from caffeine by *Pseudomonas putida* No. 352. Biosci. Biotech. Biochem. 58:2303–2304.

Ashengroph, M., and S. Ababaf. 2013. Biodecaffeination by *Pseudomonas pseudoalcaligenes* TPS8, an isolated strain from tea plantation soil. J. Sci., Islamic Republic of Iran 24:305–312.

Baker, S., S. Sahana, D. Rakshith, H. U. Kavitha, K. S. Kavitha, and S. Satish. 2012. Biodecaffeination by endophytic *Pseudomonas* sp. isolated from *Coffea arabica* L. J. Pharm. Res. 5:3654–3657.

Blecher, R. 1976. Mikrobieller Abbau von Koffein. Zbl. Bakt. Hyg., I. Abt. Orig. B 162:180–183.

Blecher, R., and F. Lingens. 1977. The metabolism of caffeine by a *Pseudomonas putida* strain. Hoppe-Seyler’s Z. Physiol. Chem. 358:807–817.

Ceja-Navarro, J. A., F. E. Vega, U. Karaoz, Z. Hao, S. Jenkins, H. C. Lim, P. Kosina, F. Infante, T. R. Northen, and E. L. Brodie. 2015. Gut microbiota mediate caffeine detoxification in the primary insect pest of coffee. Nat. Commun. 6:7618.

Dash, S. S., and S. N. Gummadi. 2006. Biodegradation of caffeine by *Pseudomonas* sp. NCIM 5235. Res. J. Microbiol. 1:115–123.

Divine, R. 2014. Isolation of bacteria from compost for potential use in biodecaffeination. Honors thesis, College of Agriculture and Life Sciences, Physical Sciences, Cornell University.

El-Mched, F., Z. Olama, and H. Holail. 2013. Optimization of the environmental and physiological factors affecting microbial caffeine degradation and its application in caffeinated products. Basic Res. J. Microbiol. 1:17–27.

Fan, F.-Y., Y. Xu, Y.-R. Liang, X.-Q. Zheng, D. Borthakur, and J.-L. Lu. 2011. Isolation and characterization of high caffeine-tolerant bacterium strains from the soil of tea garden. Afr. J. Microbiol. Res. 5:2278–2286.

Franke, W., and G. E. Hahn. 1955. Untersuchungen zum bakteriellen Purin-Abbau. II. Über den Abbau von Amino-, Oxy- und Methyl-purinen durch *Pseudomonas aeruginosa* (*B. pyocyaneum*). Hoppe Seyler’s Z. Physiol. Chem. 301:90–106

Glück, M., and F. Lingens. 1987. Studies on the microbial production of theobromine and heteroxanthine from caffeine. Appl. Microbiol. Biotechnol. 25:334–340.

Glück, M., and F. Lingens. 1988. Heteroxanthinedemethylase, a new enzyme in the degradation of caffeine by *Pseudomonas putida*. Appl. Microbiol. Biotechnol. 28:59–62.

Gokulakrishnan, S., K. Chandraraj, S. N. Gummadi. 2007. A preliminary study of caffeine degradation by *Pseudomonas* sp. GSC 1182. Int. J. Food Microbiol. 113:346–350.

Haas, G. J., and B. Stieglitz. 1980. Microbiological decaffeination of aqueous liquids. United States Patent No. 4,228,191.

Hohnloser, W., B. Osswald, and F. Lingens. 1980. Enzymological aspects of caffeine demethylation and formaldehyde oxidation by *Pseudomonas putida* C1. Hoppe-Seyler’s Z. Physiol. Chem. 361:1763–1766.

Ibrahim, S., M. Y. Shukor, M. A. Syed, W. L. W. Johari, and S. A. Ahmad. 2016a. Characterisation and growth kinetics studies of caffeine-degrading bacterium *Leifsonia* sp. strain SIU. Ann. Microbiol. 66:289–298.

Ibrahim, S., M. Y. Shukor, M. A. Syed, W. L. W. Johari, N. A. Shamaan, M. K. Sabullah, and S. A. Ahmad. 2016b. Enhanced caffeine degradation by immobilized cells of *Leifsonia* sp. stain SIU. J. Gen. Appl. Microbiol. 62:18–24.

Koide, Y., S. Nakane, and Y. Imai. 1996. Caffeine demethylate gene-containing DNA fragment and microbial process for producing 3-methyl-7-alkylxanthine. United States Patent No. 5,550,041.

Kurtzman, R.H., Jr., and S. Schwimmer. 1971. Caffeine removal from growth media by microorganisms. Experientia 27:481–482.

Madyastha, K. M., and G. R. Sridhar. 1998. A novel pathway for the metabolism of caffeine by a mixed culture consortium. Biochem. Biophys. Res. Commun. 249:178–181.

Mazzafera, P., O. Olsson, G. Sandberg. 1996. Degradation of caffeine and related methylxanthines isolated from soil under coffee cultivation. Microb. Ecol. 31:199–207.

Middelhoven, W. J., and C. M. Bakker. 1982. Degradation of caffeine by immobilized cells of *Pseudomonas putida* strain C 3024. Eur. J. Appl. Microbiol. Biotechnol. 15:214–217.

Mohapatra, B. R., N. Harris, R. Nordin, and A. Mazumder. 2006. Purification and characterization of a novel caffeine oxidase from *Alcaligenes* species. J. Biotechnol. 125:319–327.

Nayak, S., M. J. Harshitha, Maithili, C. Sampath, H. S. Anilkumar, and C. V. Rao. 2012. Isolation and characterization of caffeine degrading bacteria from coffee pulp. Indian J. Biotechnol. 11:86–91.

Nunes, F. V., and I. S. de Melo. 2006. Isolation and characterization of endophytic bacteria of coffee plants and their potential in caffeine degradation. WIT Trans. Biomed. Health 10:293–297.

Ogunseitan, O. A. 1996. Removal of caffeine in sewage by *Pseudomonas putida*: implications for water pollution index. World J. Microbiol. Biotechnol. 12:251–256.

Ogunseitan, O. A. 2002. Caffeine-inducible enzyme activity in *Pseudomonas putida* ATCC 700097. World J. Microbiol. Biotechnol. 18:423–428.

Parales, R. E., G. Sharma, X. Zhang, G. A. Subuyuj, J. T. Langner, M. E. Wright, J. L. Ditty, and S. C. Dawson. 2019. Draft genome sequence of the caffeine-degrading methylotroph *Methylorubrum populi* Pinkel. Microbiol. Resour. Announc. 8:e01300-19.

Pathak, A., S. J. Green, A. Ogram, and A. Chauhan. 2013. Draft genome sequence of *Rhodococcus opacus* strain M213 shows a diverse catabolic potential. Genome Announc.1(1):e00144-12.

Quandt, E. M., R. M. Summers, M. V. Subramanian, and J. E. Barrick, 2015. Draft genome sequence of the bacterium *Pseudomonas putida* CBB5, which can utilize caffeine as a sole carbon and nitrogen source. Genome Announc. 3(3):e00640-15.

Sarath Babu, V. R., S. Patra, M. S. Thakur, N. G. Karanth, and M. C. Varadaraj. 2005. Degradation of caffeine by *Pseudomonas alcaligenes* CFR 1708. Enzyme Microb. Technol. 37:617–624.

Sideso, O. F. P., A. C. Marvier, N. A. Katerelos, and P. W. Goodenough. 2001. The characteristics and stabilization of a caffeine demethylase enzyme complex. Int. J. Food Sci. Technol. 36:693–698.

Summers, R., J. Shao, M. B. Mock, C. L. Yu, and F. E. Vega. 2020. Draft genome of *Pseudomonas* sp. strain CES, containing the entire alkylxanthine gene cluster for caffeine breakdown. Microbiol. Resour. Announc. 9:e00484-20.

Sun, D., X. Yang, C. Zeng, B. Li, Y. Wang, C. Zhang, W. Hu, X. Li, and Z. Gao. 2020. Novel caffeine degradation gene cluster is mega-plasmid encoded in *Paraburkholderia caffeinilytica* CF1. Appl. Microbiol. Biotechnol. 104:3025–3036.

Topp, E., J. G. Hendel, Z. Lu, and R. Chapman. 2006. Biodegradation of caffeine in agricultural soils. Can. J. Soil Sci. 86:533–544.

Watahiki, S., and N. Kimura. 2017. Draft genome sequence of a caffeine-utilizing bacterium, *Cupriavidus* sp. strain D384. Genome Announc. 5:e00370-17.

Woolfolk, C. A. 1975. Metabolism of *N*-methylpurines by a *Pseudomonas putida* strain isolated by enrichment on caffeine as the sole source of carbon and nitrogen. J. Bacteriol. 123:1088–1106.

Yamaoka-Yano, D. M., and P. Mazzafera. 1998. Degradation of caffeine by *Pseudomonas putida* isolated from soil under coffee cultivation. Allelopathy J. 5:23–34.

Yamaoka-Yano, D. M., and P. Mazzafera. 1999. Catabolism of caffeine and purification of a xanthine oxidase responsible for methyluric acids production in *Pseudomonas putida* L. Rev. Microbiol. (Brazil) 30:62–70.

Yu, C. L., Y. Kale, S. Gopishetty, T. M. Louie, and M. Subramanian. 2008. A novel caffeine dehydrogenase inn *Pseudomonas* sp. strain CBB1 oxidizes caffeine to trimethyluric acid. J. Bacteriol. 190:772–776.

Yu, C. L., T. M. Louie, R. Summers, Y. Kale, S. Gopishetty, and M. Subramanian. 2009. Two distinct pathways for metabolism of theophylline and caffeine are coexpressed in *Pseudomonas putida* CBB5. J. Bacteriol. 191:4624–4632.

1. The authors mention that the bacteria convert some methylxanthines to methyluric acids, but that the methyluric acids don't break down any further. [↑](#footnote-ref-1)
