# Supplementary material for: Cultivation and Genome Sequencing of Bacteria Isolated From the Coffee Berry Borer (Hypothenemus hampei), With Emphasis on the Role of Caffeine Degradation
Source: Front Microbiol. 2021 Apr 6;12:644768. doi: 10.3389/fmicb.2021.644768 (PMC8055839; doi:10.3389/fmicb.2021.644768)
Supplement: Supplementary Table 2 — Assembly statistics for 50 bacterial strains isolated from the coffee berry borer. [file Table_2.docx]

**Supplementary Table 2.** Assembly statistics for 50 bacterial strains isolated from the coffee berry borer.

| **Isolate** | **Illumina Sequence ID** | **GC%** | **Contigs**  **(>200 bp)** | **Total length**  **(bp)** | **N50** | **Avg. read coverage** | **GenBank**  **Accession #** |
| --- | --- | --- | --- | --- | --- | --- | --- |
| **S40** | *Acinetobacter* sp. S40 | 39.48% | 53 | 3526298 | 246709 | 264.278 | JACVTU000000000 |
| **S54** | *Acinetobacter* sp. S54 | 39.57% | 44 | 3547076 | 261476 | 168.155 | JACVUI000000000 |
| **S55** | *Acinetobacter* sp. S55 | 39.58% | 50 | 3545689 | 142608 | 111.769 | JACVUJ000000000 |
| **S34** | *Bacillus aryabhattai* | 41.61% | 2008 | 6185119 | 452749 | 107.188 | JACVTQ000000000 |
| **S35** | *Bacillus aryabhattai* | 37.40% | 75 | 5822166 | 821340 | 184.255 | JACVTR000000000 |
| **S56** | *Bacillus cereus group* | 34.80% | 54 | 5638421 | 317523 | 174.417 | JACVUK000000000 |
| **S74** | *Bacillus cereus group* | 35.12% | 422 | 5305448 | 32672 | 15.038 | JACVUZ000000000 |
| **S29** | *Bacillus* sp. S29 | 35.07% | 53 | 5343748 | 370736 | 166.959 | JACVTN000000000 |
| **S70** | *Bacillus* sp. S70 | 35.10% | 65 | 5350084 | 309358 | 170.023 | JACVUV000000000 |
| **S71** | *Bacillus* sp. S71 | 35.07% | 44 | 5344799 | 450691 | 119.823 | JACVUW000000000 |
| **S72** | *Bacillus* sp. S72 | 35.08% | 55 | 5346887 | 309147 | 259.752 | JACVUX000000000 |
| **S73** | *Bacillus* sp. S73 | 35.07% | 48 | 5345259 | 309429 | 191.153 | JACVUY000000000 |
| **S65** | *Delftia lacustris* | 66.63% | 144 | 6655985 | 107377 | 115.307 | JACVUR000000000 |
| **S66** | *Delftia lacustris* | 66.63% | 158 | 6658550 | 101442 | 95.076 | JACVUS000000000 |
| **S67** | *Delftia lacustris* | 66.63% | 148 | 6657423 | 116852 | 129.709 | JACVUT000000000 |
| **S52** | *Enterococcus* sp. S52 | 41.77% | 111 | 3914349 | 238623 | 71.371 | JACVUG000000000 |
| **S53** | *Enterococcus* sp. S53 | 41.77% | 96 | 3913592 | 239435 | 153.698 | JACVUH000000000 |
| **S76** | *Enterococcus* sp. S76 | 41.77% | 98 | 3913774 | 261963 | 197.356 | JACVVB000000000 |
| **S77** | *Enterococcus* sp. S77 | 41.77% | 101 | 3912865 | 238623 | 94.186 | JACVVC000000000 |
| **S38** | *Erwinia* sp. S38 | 55.08% | 100 | 5889904 | 195743 | 129.23 | JACVTS000000000 |
| **S43** | *Erwinia* sp. S43 | 55.37% | 97 | 5849522 | 190707 | 182.95 | JACVTX000000000 |
| **S59** | *Erwinia* sp. S59 | 53.56% | 58 | 5570918 | 210063 | 130.865 | JACVUN000000000 |
| **S63** | *Erwinia* sp. S63 | 53.60% | 62 | 5856603 | 255009 | 106.071 | JACVUP000000000 |
| **S69** | *Klebsiella oxytoca* | 54.81% | 130 | 6164919 | 174227 | 84.652 | JACVUU000000000 |
| **S57** | *Kosakonia cowanii* | 55.72% | 57 | 5072378 | 183484 | 156.457 | JACVUL000000000 |
| **S58** | *Kosakonia cowanii* | 55.71% | 62 | 5072703 | 160919 | 116.979 | JACVUM000000000 |
| **S42** | *Kosakonia cowanii* | 55.44% | 140 | 5388599 | 184344 | 196.759 | JACVTW000000000 |
| **S47** | *Lactococcus* sp. S47 | 34.82% | 34 | 2359028 | 241089 | 259.846 | JACVUB000000000 |
| **S64** | *Lactococcus* sp. S64 | 34.77% | 49 | 2479731 | 250959 | 251.867 | JACVUQ000000000 |
| **S50** | *Leuconostoc* sp. S50 | 38.82% | 56 | 2245379 | 144114 | 324.917 | JACVUE000000000 |
| **S51** | *Leuconostoc* sp. S51 | 38.83% | 110 | 2265576 | 122265 | 160.358 | JACVUF000000000 |
| **S45** | *Ochrobactrum* sp. S45 | 53.55% | 39 | 5320973 | 426359 | 147.296 | JACVTZ000000000 |
| **S46** | *Ochrobactrum* sp. S46 | 53.55% | 46 | 5321781 | 426359 | 166.082 | JACVUA000000000 |
| **S28** | *Paenibacillus* sp. S28 | 52.99% | 70 | 7483663 | 435767 | 55.764 | JACVTM000000000 |
| **S61** | *Pantoea* sp. S61 | 53.27% | 83 | 6343430 | 236021 | 101.515 | JACVUO000000000 |
| **S62** | *Pantoea* sp. S62 | 54.99% | 89 | 5300361 | 238713 | 120.599 | JACVVI000000000 |
| **S68** | *Pseudomonas aeruginosa* | 66.42% | 133 | 6391454 | 97245 | 72.015 | JACVZF000000000 |
| **S33** | *Pseudomonas aeruginosa* | 66.42% | 113 | 6393504 | 125735 | 111.034 | JACVTP000000000 |
| **S36** | *Pseudomonas parafulva* | 62.29% | 138 | 5203739 | 91183 | 114.139 | JACVVG000000000 |
| **S30** | *Pseudomonas* sp. S30 | 64.00% | 68 | 5474368 | 208400 | 107.912 | JACVTO000000000 |
| **S31** | *Pseudomonas* sp. S31 | 63.54% | 114 | 6374075 | 118279 | 119.273 | JACVVE000000000 |
| **S32** | *Pseudomonas* sp. S32 | 62.22% | 142 | 5260104 | 100596 | 127.611 | JACVVF000000000 |
| **S37** | *Pseudomonas* sp. S37 | 62.34% | 271 | 6706203 | 44983 | 166.139 | JACVZC000000000 |
| **S44** | *Pseudomonas* sp. S44 | 63.35% | 89 | 5693087 | 126837 | 86.712 | JACVTY000000000 |
| **S60** | *Pseudomonas* sp. S60 | 62.27% | 145 | 5432536 | 99554 | 139.945 | JACVVH000000000 |
| **S75** | *Pseudomonas* sp. S75 | 63.90% | 87 | 5555732 | 150216 | 131.588 | JACVVA000000000 |
| **S39** | *Stenotrophomonas* sp. S39 | 66.68% | 235 | 4916522 | 42065 | 196.231 | JACVTT000000000 |
| **S41** | *Stenotrophomonas* sp. S41 | 67.15% | 252 | 4632962 | 35117 | 188.716 | JACVTV000000000 |
| **S48** | *Stenotrophomonas* sp. S48 | 66.94% | 192 | 4456577 | 41395 | 202.947 | JACVUC000000000 |
| **S49** | *Stenotrophomonas* sp. S49 | 66.94% | 190 | 4455747 | 36660 | 160.048 | JACVUD000000000 |
