# Supplementary material for: Cultivation and Genome Sequencing of Bacteria Isolated From the Coffee Berry Borer (Hypothenemus hampei), With Emphasis on the Role of Caffeine Degradation
Source: Front Microbiol. 2021 Apr 6;12:644768. doi: 10.3389/fmicb.2021.644768 (PMC8055839; doi:10.3389/fmicb.2021.644768)
Supplement: Supplementary Table 3 — Bacteria isolated from the coffee berry borer, indicating which strains might have C-8 oxidation activity (cdhA, cdhB, cdhC, tmuM, tmuH, tmuD), coffee berry borer source used for bacterial isolation (eggs, frass, heads, larvae), non-macerated (whole) or macerated tissue, sterile (St) or non-sterile (NSt), sampling site, GenBank accession numbers, and average nucleotide identity (ANI; Chun et al., 2018) analysis. All insects were field collected, except “Beltsville, MD”, which were reared in the laboratory (see column labeled “Sampling Site”). [file Table_3.docx]

**Supplementary Table 3.** Bacteria isolated from the coffee berry borer, indicating which strains might have C-8 oxidation activity (*cdhA*, *cdhB*, *cdhC*, *tmuM*, *tmuH*, *tmuD*), coffee berry borer source used for bacterial isolation (eggs, frass, heads, larvae), non-macerated (whole) or macerated tissue, sterile (St) or non-sterile (NSt), sampling site, GenBank accession numbers, and average nucleotide identity (ANI; Chun et al. 2018) analysis. All insects were field collected, except “Beltsville, MD”, which were reared in the laboratory (see column labeled “Sampling Site”).

| **Isolate** | ***Illumina Sequence ID*** | ***cdhA*** | ***cdhB*** | ***cdhC*** | ***tmuM*** | ***tmuH*** | ***tmuD*** | **Source** | **Whole/Macerated** | **St/NSt** | **Sampling Site** | **GenBank Accession #** | **ANI** |
| --- | --- | --- | --- | --- | --- | --- | --- | --- | --- | --- | --- | --- | --- |
| **S40** | *Acinetobacter* sp. S40 | CdhA 7E-46 |  | CdhC 3E-25 | TmuM 2E-87 |  | TmuD 3E-15 | Eggs | Whole | St | Mexico | JACVTU000000000 | 84.19 |
| **S54** | *Acinetobacter* sp. S54 | CdhA 1E-46 |  | CdhC 5E-25 | TmuM 2E-87 |  | TmuD 4E-15 | Eggs | Whole | NSt | Oahu, Hawai'i | JACVUI000000000 | 84.20 |
| **S55** | *Acinetobacter* sp. S55 | CdhA 1E-46 |  | CdhC 5E-25 | TmuM 2E-87 |  | TmuD 4E-15 | Eggs | Whole | NSt | Oahu, Hawai'i | JACVUJ000000000 | 84.16 |
| **S34** | *Bacillus aryabhattai* | CdhA 2E-56 | CdhB 1E-15 | CdhC 3E-36 | TmuM 2E-14 | TmuH 3E-22 | TmuD 2E-35 | Eggs | Macerated | St | Mexico | JACVTQ000000000 | 99.53 |
| **S35** | *Bacillus aryabhattai* | CdhA 4E-59 | CdhB 4E-16 | CdhC 2E-36 | TmuM 3E-13 | TmuH 9E-21 | TmuD 2E-35 | Frass | N/A | N/A | Beltsville, Maryland | JACVTR000000000 | 95.93 |
| **S56** | *Bacillus cereus group* |  |  |  |  |  |  | Eggs | Whole | NSt | Oahu, Hawai'i | JACVUK000000000 | 97.36 |
| **S74** | *Bacillus cereus group* |  |  |  | TmuM 3E-18 |  |  | Heads | Macerated | St | Beltsville, Maryland | JACVUZ000000000 | 96.54 |
| **S29** | *Bacillus* sp. S29 |  |  |  | TmuM 3E-18 |  |  | Heads | Macerated | St | Beltsville, Maryland | JACVTN000000000 | 91.48 |
| **S70** | *Bacillus* sp. S70 |  |  |  | TmuM 3E-18 |  |  | Heads | Macerated | St | Beltsville, Maryland | JACVUV000000000 | 91.49 |
| **S71** | *Bacillus* sp. S71 |  |  |  | TmuM 3E-18 |  |  | Heads | Macerated | St | Beltsville, Maryland | JACVUW000000000 | 91.45 |
| **S72** | *Bacillus* sp. S72 |  |  |  | TmuM 3E-18 |  |  | Heads | Macerated | St | Beltsville, Maryland | JACVUX000000000 | 91.49 |
| **S73** | *Bacillus* sp. S73 |  |  |  | TmuM 3E-18 |  |  | Heads | Macerated | St | Beltsville, Maryland | JACVUY000000000 | 91.47 |
| **S65** | *Delftia lacustris* | CdhA 6E-138 | CdhB 8E-38 | CdhC 9E-55 | TmuM 2E-36 | TmuH 2E-23 | TmuM 3E-24 | Heads | Macerated | St | K'au, Hawai'i | JACVUR000000000 | 95.08 |
| **S66** | *Delftia lacustris* | CdhA 6E-138 | CdhB 8E-38 | CdhC 9E-55 | TmuM 2E-36 | TmuH 2E-23 | TmuM 3E-24 | Heads | Macerated | St | K'au, Hawai'i | JACVUS000000000 | 95.10 |
| **S67** | *Delftia lacustris* | CdhA 6E-138 | CdhB 8E-38 | CdhC 9E-55 | TmuM 2E-36 | TmuH 2E-23 | TmuM 3E-24 | Heads | Macerated | St | K'au, Hawai'i | JACVUT000000000 | 94.97 |
| **S52** | *Enterococcus* sp. S52 |  |  |  |  |  |  | Heads | Macerated | St | Mexico | JACVUG000000000 | 75.94 |
| **S53** | *Enterococcus* sp. S53 |  |  |  |  |  |  | Heads | Macerated | St | Mexico | JACVUH000000000 | 76.02 |
| **S76** | *Enterococcus* sp. S76 |  |  |  |  |  |  | Heads | Macerated | St | Mexico | JACVVB000000000 | 75.91 |
| **S77** | *Enterococcus* sp. S77 |  |  |  |  |  |  | Heads | Macerated | St | Mexico | JACVVC000000000 | 76.07 |
| **S38** | *Erwinia* sp. S38 | CdhA 2E-37 | CdhB 8E-12 | CdhC 7E-38 | TmuM 4E-89 |  | TmuD 4E-18 | Eggs | Whole | St | Mexico | JACVTS000000000 | 79.12 |
| **S43** | *Erwinia* sp. S43 | CdhA 4E-12 |  | CdhC 2E-29 | TmuM 3E-90 |  | TmuD 3E-18 | Larvae | Whole | NSt | Mexico | JACVTX000000000 | 79.07 |
| **S59** | *Erwinia* sp. S59 | CdhA 1E-55 | CdhB 8E-12 | CdhC 7E-38 | TmuM 3E-94 |  | TmuD 2E-16 | Heads | Macerated | NSt | Holualoa, Hawai'i | JACVUN000000000 | 75.27 |
| **S63** | *Erwinia* sp. S63 | CdhA 6E-55 | CdhB 8E-12 | CdhC 7E-38 | TmuM 2E-94 |  | TmuD 8E-18 | Heads | Macerated | St | K'au, Hawai'i | JACVUP000000000 | 75.50 |
| **S69** | *Klebsiella oxytoca* | CdhA 6E-65 |  | CdhC 1E-47 | TmuM 3E-90 |  | TmuM 6E-16 | Heads | Macerated | St | Mexico | JACVUU000000000 | 99.10 |
| **S57** | *Kosakonia cowanii* | CdhA 2E-59 | CdhB 6E-10 | CdhC 5E-48 | TmuM 1E-10 |  | TmuD 1E-19 | Eggs | Whole | St | Oahu, Hawai'i | JACVUL000000000 | 96.86 |
| **S58** | *Kosakonia cowanii* | CdhA 2E-59 | CdhB 6E-10 | CdhC 5E-48 | TmuM 1E-10 |  | TmuD 1E-19 | Eggs | Whole | St | Oahu, Hawai'i | JACVUM000000000 | 96.78 |
| **S42** | *Kosakonia cowanii* | CdhA 8E-61 | CdhB 6E-10 | CdhC 2E-48 | TmuM 4E-10 |  | TmuD 1E-19 | Heads | Macerated | St | Mexico | JACVTW000000000 | 96.82 |
| **S47** | *Lactococcus* sp. S47 |  |  |  |  |  |  | Heads | Macerated | St | Mexico | JACVUB000000000 | 86.21 |
| **S64** | *Lactococcus* sp. S64 |  |  |  |  |  |  | Frass-berry | N/A | N/A | K'au, Hawai'i | JACVUQ000000000 | 86.10 |
| **S50** | *Leuconostoc* sp. S50 |  |  |  |  |  |  | Heads | Macerated | St | Mexico | JACVUE000000000 | 75.59 |
| **S51** | *Leuconostoc* sp. S51 |  |  |  |  |  |  | Heads | Macerated | St | Mexico | JACVUF000000000 | 75.61 |
| **S45** | *Ochrobactrum* sp. S45 | CdhA 2E-52 |  | CdhC 1E-26 | TmuM 3E-25 | TmuH 2E-30 | TmuD 5E-34 | Eggs | Macerated | St | Mexico | JACVTZ000000000 | 83.82 |
| **S46** | *Ochrobactrum* sp. S46 | CdhA 2E-52 |  | CdhC 1E-26 | TmuM 3E-25 | TmuH 2E-30 | TmuD 5E-34 | Eggs | Macerated | St | Mexico | JACVUA000000000 | 83.99 |
| **S28** | *Paenibacillus* sp. S28 | CdhA 5E-64 | CdhB 7E-12 | CdhC 9E-44 | TmuM 2E-14 | TmuH 2E-19 | TmuD 1E-30 | Eggs | Macerated | St | Beltsville, Maryland | JACVTM000000000 | 68.92 |
| **S61** | *Pantoea* sp. S61 | CdhA 1E-55 | CdhB 8E-12 | CdhC 7E-38 | TmuM 2E-94 |  | TmuM 3E-16 | Heads | Macerated | St | Holualoa, Hawai'i | JACVUO000000000 | 86.23 |
| **S62** | *Pantoea* sp. S62 | CdhA 6E-54 | CdhB 1E-23 |  | TmuM 4E-87 | TmuH 7E-13 | TmuD 5E-15 | Heads | Macerated | NSt | K'au, Hawai'i | JACVVI000000000 | 79.26 |
| **S68** | *Pseudomonas aeruginosa* | CdhA 1E-50 | CdhB 2E-14 | CdhC 1E-38 | TmuM 2E-60 | TmuH 3E-25 | TmuD 7E-27 | Heads | Macerated | St | Mexico | JACVZF000000000 | 99.42 |
| **S33** | *Pseudomonas aeruginosa* | CdhA 1E-50 | CdhB 2E-14 | CdhC 1E-38 | TmuM 2E-60 | TmuH 3E-25 | TmuD 7E-27 | Eggs | Macerated | St | Mexico | JACVTP000000000 | 99.44 |
| **S36** | *Pseudomonas parafulva* | CdhA 3E-53 | CdhB 2E-13 | CdhC 1E-38 |  | TmuH 6E-23 | TmuD 2E-32 | Frass | N/A | N/A | Beltsville, Maryland | JACVVG000000000 | 98.05 |
| **S30** | *Pseudomonas* sp. S30 | CdhA 1E-55 | CdhB 2E-14 | CdhC 3E-42 |  | TmuH 6E-20 | TmuD 5E-31 | Heads | Macerated | St | Beltsville, Maryland | JACVTO000000000 | 82.20 |
| **S31** | *Pseudomonas* sp. S31 | CdhA 3E-53 | CdhB 4E-13 | CdhC 1E-39 | TmuM 3E-36 | TmuH 5E-23 | TmuD 3E-32 | Eggs | Whole | NSt | Mexico | JACVVE000000000 | 86.15 |
| **S32** | *Pseudomonas* sp. S32 | CdhA 3E-53 | CdhB 1E-13 | CdhC 1E-38 |  | TmuH 6E-23 | TmuD 2E-32 | Eggs | Whole | St | Mexico | JACVVF000000000 | 84.66 |
| **S37** | *Pseudomonas* sp. S37 | CdhA 5E-53 |  | CdhC 2E-34 | TmuM 5E-41 | TmuH 3E-22 | TmuD 2E-30 | Eggs | Whole | St | Mexico | JACVZC000000000 | 89.53 |
| **S44** | *Pseudomonas* sp. S44 | CdhA 2E-53 | CdhB 8E-14 | CdhC 4E-38 |  | TmuH 2E-23 | TmuD 4E-31 | Eggs | Whole | NSt | Mexico | JACVTY000000000 | 83.88 |
| **S60** | *Pseudomonas* sp. S60 | CdhA 3E-53 | CdhB 1E-13 | CdhC 7E-40 |  | TmuH 6E-23 | TmuD 3E-32 | Heads | Macerated | St | Holualoa, Hawai'i | JACVVH000000000 | 84.78 |
| **S75** | *Pseudomonas* sp. S75 | CdhA 1E-55 | CdhB 2E-14 | CdhC 3E-42 |  | TmuH 6E-20 | TmuD 5E-31 | Heads | Macerated | St | Beltsville, Maryland | JACVVA000000000 | 82.14 |
| **S39** | *Stenotrophomonas* sp. S39 | CdhA 5E-40 | CdhB 8E-12 | CdhC 3E-40 | TmuM 5E-19 |  |  | Eggs | Whole | St | Mexico | JACVTT000000000 | 87.79 |
| **S41** | *Stenotrophomonas* sp. S41 | CdhA 6E-38 | CdhB 1E-10 | CdhC 7E-41 | TmuM 5E-42 |  |  | Heads | Macerated | St | Mexico | JACVTV000000000 | 87.72 |
| **S48** | *Stenotrophomonas* sp. S48 |  |  |  | TmuM 3E-22 |  |  | Heads | Macerated | St | Mexico | JACVUC000000000 | 86.77 |
| **S49** | *Stenotrophomonas* sp. S49 |  |  |  | TmuM 3E-22 |  |  | Heads | Macerated | St | Mexico | JACVUD000000000 | 86.76 |
